# The Viscoelastic Behavior of Legume Protein Emulsion Gels—The Effect of Heating Temperature and Oil Content on Viscoelasticity, the Degree of Networking, and the Microstructure

**DOI:** 10.3390/foods13233875

**Published:** 2024-11-29

**Authors:** Lena Johanna Langendörfer, Elizaveta Guseva, Peter Bauermann, Andreas Schubert, Oliver Hensel, Mamadou Diakité

**Affiliations:** 1 Faculty of Food Technology, University of Applied Science Fulda, Leipziger Str. 123, 36037 Fulda, Germany; 2 Specialty Additives—RD&I Coating Additives—Particle Design, EVONIK Operations GmbH, Rodenbacher Chaussee 4, 63457 Hanau-Wolfgang, Germany; 3 Anton Paar Germany GmbH, Hellmuth-Hirth-Strasse 6, 73760 Ostfildern-Scharnhausen, Germany; 4 Faculty of Organic Agricultural Science, University of Kassel, Nordbahnhofstraße 1a, 37213 Witzenhausen, Germany

**Keywords:** legume emulsion gel, heating, oil, viscoelasticity, microstructure

## Abstract

Legume proteins are increasingly used in structuring various foods under the influence of heating and stirring energy. Based on available studies, this structuring potential is not yet fully understood. This raises the question of the suitability of legume isolates and concentrates for structuring in emulsion gels and the effect of heat and oil on the gel properties. In this study, soy- and pea-based suspensions and emulsions were prepared with the least gelling concentration using different oil concentrations (0%, 7.5%, 15%, 22.5%, and 30%). The viscoelastic properties were measured before and after heating cycles (65 °C and 95 °C). Scanning electron microscopy images complemented the results. All gels measured showed viscoelastic solid behavior. Thermal treatment showed a positive effect on the gel properties for most samples, especially for concentrates (reduction in the loss factor and networking factor > 1). The concentrates showed much higher networking factors and tighter cross-linking than the isolates. The rheological and microstructural properties of the emulsion gels are influenced by a number of factors, such as carbohydrate content, protein chemistry, the protein purification method, and initial viscosity. Moreover, the influence of oil on the rheological properties depends on the material used and whether oil droplets act as an active or inactive filler.

## 1. Introduction

Soybeans and peas are used in various alternative food applications due to their good availability, low cost, and process functionality [1]. Soy was the pioneer in terms of alternative plant proteins, but over a ten-year period, it has become clear that soy has declined, and pea has become more popular [2].

Both are rich in protein, and their amino acid composition is slightly different [3] due to the influence of the origin and purification method [4]. In terms of their chemical structure, globulins form the largest part of the quaternary structure (90% in soy and 50–60% in pea) [5]. They exhibit gel-forming properties when exposed to heat [6]. Therefore, concentrates and isolates made of soy and pea are being increasingly used in a variety of plant-based food applications.

If the proteins are gelled (heat treatment, acidification, or enzyme treatment) in an emulsion with low or moderate oil content and well-separated oil droplets, a viscoelastic emulsion gel is formed [7,8]. Emulsion gels can be divided into two structural arrangements: the emulsion-filled protein gel and the protein-stabilized emulsion gel. The former is a particle-filled solid whose solid-like rheological properties are predominantly determined by the network properties of the spatially continuous matrix. The latter is a type of particulate gel whose rheological properties are mainly determined by the properties of the network of aggregated emulsion droplets [9].

Rheological and microstructural data can be used to describe the molecular organization and structural properties of complex and viscoelastic materials like emulsion systems. The most commonly used rheological model to describe viscoelastic behavior is the linear viscoelastic model with the linear viscoelastic range (LVR) and the key parameters G′ (elastic fraction) and G″ (viscous fraction) within this range [10,11]. In the viscoelastic range, a distinction is made between viscoelastic liquid (G″ > G′) and solid (G′ > G″).

Few studies have investigated the rheological and microstructural properties of legume emulsion gels. Kim et al. (2001) investigated the influence of different volume fractions of oil droplets and sodium chloride (0.2 M) on the rheological properties of soy protein isolate emulsion gels during the heating process. During an applied temperature cycle, the G′ values increased strongly after the heating and cooling phases, and the loss tan δ decreased compared to the beginning of the cycle. Furthermore, an increasing oil concentration led to both an increasing G′ module and a decreasing tan δ [12].

Gu et al. (2009) studied the influence of different oils (sunflower, soybean, and palm stearin) at a neutral pH on the rheological properties of soy protein isolate emulsion gels. As the oil concentration increased, both G′ and G″ increased, and the gels became more elastic. However, the values at 5% oil (sunflower and soybean) content showed no significant differences from the soy protein isolate hydrogel. No significant differences were found between sunflower and soybean oils [13].

Kornet et al. (2022) investigated the rheological and microstructural differences between emulsion gels of pea protein isolates obtained via isoelectric precipitation or diafiltration and rapeseed oil at varying pH levels and oil concentrations. At pH 7, the pea protein isolate obtained using isoelectric precipitation formed weaker and more heterogeneous gels than the pea protein isolate obtained using diafiltration. Increasing the oil concentration did not lead to a stronger gel structure, and oil acted as an inert filler [14].

The number of studies focusing on plant-based emulsion gels is limited. In addition, there is no direct comparison between different legumes, such as soya and pea, or between isolates and concentrates to assess the suitability of more sustainable and alternative raw materials. Consequently, the potential of legume raw materials as texturing agents in emulsion gels has not been fully exploited. Therefore, the potential of legume isolates and concentrates (soy and pea) as texturing agents in heat-induced emulsion gels was investigated and compared.

The aim of this study was to investigate the gelling properties of the raw materials used (macronutrient content and amino acid composition) by determining their rheological and microstructural properties. Furthermore, the stability and elasticity of the different hydro- and emulsion gels were determined, and the influence of oil on the rheological and microstructural properties was measured.

## 2. Materials and Methods

### 2.1. Materials

Soy protein isolate (SPI, Vegacon 90 KK, EUROSOY (Hamburg, Germany), acid precipitation), pea protein isolate (PPI, Pisane M9, Cosucra (Warcoing, Belgium), soluble protein thermal treatment), soy protein concentrate (SPC, Vegacon 70, EUROSOY (Hamburg, Germany), alcohol soaking and extruding), and sunflower oil (Chemiekontor.de GmbH, Mannheim, Germany) were purchased. Herba ingredients (Wormer, The Netherlands) kindly provided the pea protein concentrate (PPC, YP 55-010, dry milling). The nutritional values of the individual protein raw materials and the amino acid composition are shown in Table 1 and Table 2.

### 2.2. Methods

#### 2.2.1. Preparation of Suspensions and Emulsions

An amount of 25 g of the emulsions was prepared in a 50 mL sealable tube. The emulsions were prepared with the least gelling concentration (method description and results are shown in the Appendix A), protein powder concentration, deionized water, and the following sunflower oil concentration: 0%, 7.5%, 15%, 22.5%, and 30%. The suspensions and emulsions were mixed for 30 s using a shaker (IKA Vibrofix VF1 Electronic, Staufen, Germany) at 2500 rpm, followed by homogenization with an Ultra Turrax IKA T18 (Staufen, Germany) basic at 7000 rpm for 1 min.

#### 2.2.2. Rheological Measurement

Rheological measurements were carried out using an MCR 702e axial–torsional dynamic mechanical analyzer (DMA) and a rheometer (Anton Paar, Graz, Austria). The storage and loss moduli of the samples were determined using a concentric cylinder (CC27, Anton Paar, Austria). Samples were subjected to a temperature cycle starting at 25 °C, as follows: Step 1: heating (4 °C/min); Step 2: holding at 65 °C or 95 °C for 10 min; Step 3: cooling (4 °C/min). Before heating and after cooling, an amplitude test was performed in controlled shear stress mode from a 0.01% to 160% shear rate. During the temperature cycle, a shear deformation of 5% was applied to the sample. The solvent trap used was developed by Evonik Operations GmbH (Hanau, Germany) under the description ‘Water trap’ according to Evonik Coating Additives’ by Mr. Peter Bauermann. This solvent trap, colloquially known as the Bauermann trap, offers the possibility of carrying out a friction-free measurement by means of coaxial cylinder geometry using the siphon effect in a system that is virtually sealed off from the environment. This also allows measurements to be carried out with the rheometer in oscillation. The linear viscoelastic region (LVR) limit was calculated with a tolerance range of 3%. The loss factor (G″/G′) and maximum G′ in the LVR were also determined. In order to better quantify cross-linking, a networking factor was determined and calculated as follows:

(1) $$\mathrm{networking}\;\mathrm{factor}=\frac{G_{\max\;after\;heating}^{\prime }}{G_{\max\;before\;heating}^{\prime }}$$

where $G_{\max\;before\;heating}^{\prime }$ is the maximum storage module in the LVE area before the heating cycle (Pa), and $G_{\max\;after\;heating}^{\prime }$ is the maximum storage module in the LVE area after the heating cycle (Pa)

#### 2.2.3. Scanning Electron Microscopy (SEM)

Suspensions and emulsions were prepared as described above for the microstructural analyses. The tubes were then placed in a water bath at 95 °C for 60 min. Immediately after heating, the tubes were cooled at 5–7 °C overnight in a cold storage room. Samples were taken by cutting into the gel with a knife. The gel sample was discarded toward the wall. Two clear cuts were then made to remove a disc of gel. The gel sample was then cut again from all sides at the tip of the knife in order to obtain clear-cut edges. The sample was then transferred to an Evo 25 (Carl Zeiss AG, Jena, Germany) scanning electron microscope, and images were taken with a Cascade Current Detector (C2D) at the triple point of water. The extra high tension (EHT) in kilovolts (kV), the working distance (WD), the I Probe (electron beam width) in pico ampere (pA), and the width (width of the measured sample section) varied, depending on the sample. The different rheological properties resulted in different surface structures that were analyzed microscopically. The settings were adjusted to obtain the best image quality. The settings are shown in Table 3.

#### 2.2.4. Statistical Analysis

All measurements were performed three times. The trend lines and R^2^ were calculated with MATLAB R2022a in order to better visualize the influence of oil.

## 3. Results

### 3.1. Viscoelastic Behavior

Rheological tests with variations in oil contents and temperatures were carried out, and their effects on the viscoelastic behavior were analyzed. To evaluate the viscoelasticity, the loss factor and LVR limit were used. All measuring curves can be found in the Supplementary Material, and the individual results for the viscoelasticity parameters are discussed in the following sections.

#### 3.1.1. Loss Factor

**Figure 1:**
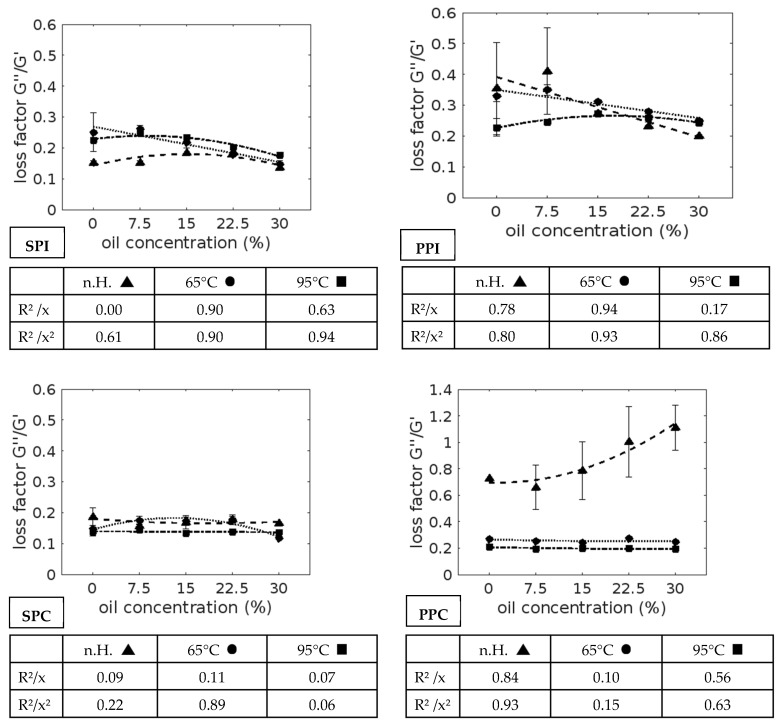
Loss factor. SPI—soy protein isolate, PPI—pea protein isolate, SPC—soy protein concentrate, PPC—pea protein concentrate, n.H.—no heating, 65 °C—after 65 °C heating cycle, 95 °C—after 95 °C heating cycle, R^2^—measure of certainty, x—linear function, x^2^—second-degree polynomial function, dashes and dots—trend line without heating cycle, dashed line—trend line after 65 °C heating cycle, dotted line—trend line after 95 °C heating cycle.

In Figure 1, the loss factor of the different samples is illustrated.

All samples showed viscoelastic solid behavior (loss factor < 1), except for PPC with 22.50% and 30.00% (loss factor > 1; viscoelastic fluid behavior) without heating. In addition, the suspension and emulsions of PPC generally have higher loss factors compared to all other samples.

Heating to 95 °C tends to reduce the loss factor. This effect is only partially visible at 65 °C. For SPI and PPI with 22.50% and 30.00%, the tendency to reduce the loss factor during the heating process is not visible. Compared to other gels, SPC generally has lower loss factors after the 95 °C heating cycle.

The influence of oil on the gel’s loss factor can be described using the approximate functions (linear and second-degree polynomials) shown in Figure 1. In the unheated samples, oil has a linear negative effect on the loss factor of pea protein isolate (R^2^ = 0.78) and only a small effect on soy protein. For PPC, an increase in the oil concentration results in a decreasing loss factor. This relationship is approximately linear (R^2^ = 0.84) but is better described using a second-degree polynomial function (R^2^ = 0.93) because the loss factor increases only slightly at a low oil concentration of 7.50%.

Heating to 65 °C changes the trend for SPI from a weak effect without heating to a positive correlation as the oil concentration increases. PPI at 65 °C also shows a positive correlation. For PPC, the gel’s loss factor is not significantly affected, as the oil concentration is increased after heating to 65 °C or 95 °C. For SPC at 95 °C, only a small effect is visible. For SPI/PPI at 95 °C and SPC at 65 °C, there is a polynomial curve with an initial positive effect and then a negative effect on the gel’s loss factor due to increasing oil concentrations.

#### 3.1.2. LVR Limit

**Figure 2:**
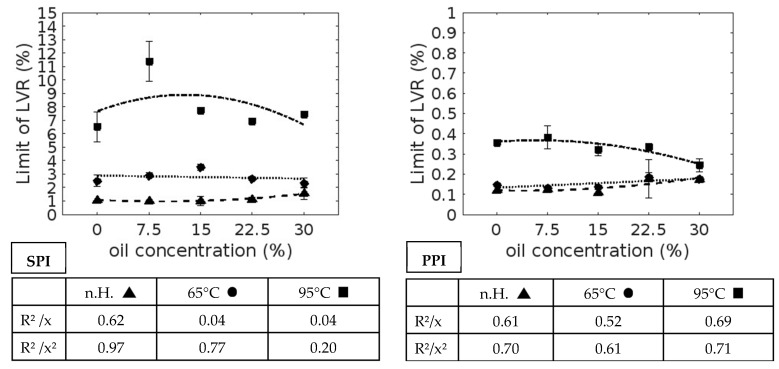

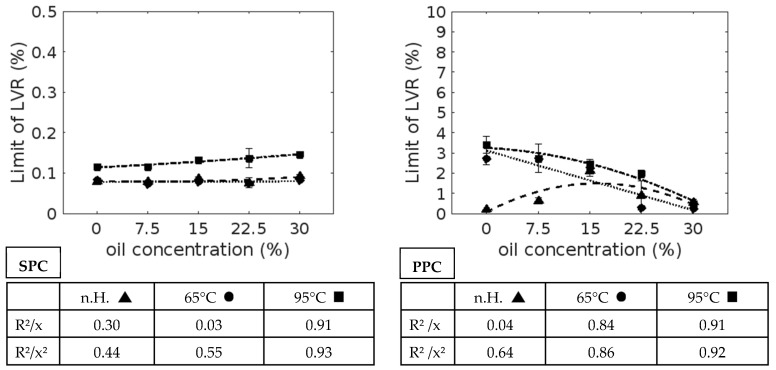
LVR limit. SPI—soy protein isolate, PPI—pea protein isolate, SPC—soy protein concentrate, PPC—pea protein concentrate, n.H.—no heating, 65 °C—after 65 °C heating cycle, 95 °C—after 95 °C heating cycle, R^2^—measure of certainty, x—linear function, x^2^—second-degree polynomial function, line of dashes and dots—trend line without heating cycle, dashed line—trend line after 65 °C heating cycle, dotted line—trend line after 95 °C heating cycle.

In addition to the gel’s loss factor, stability against shear also plays an important role in accurately describing its viscoelastic properties. The LVR limits are shown in Figure 2 below.

When the LVR limits are considered, it is noticeable that SPI achieved the highest values (1.01 (±0.05)–10.93 (±1.83), followed by PPC (0.22 (±0.22)–3.39 (±0.42)) and PPI (0.11 (±0.22)–0.38 (±0.06)). SPC showed, by far, the lowest stability (0.07 (±0.00)–0.15 (±0.00).

In addition, resistance to mechanical stress increases with heating, and a temperature increase from 65 °C to 95 °C also has a positive effect, with the exception of PPC (22.50% and 30.00% oil).

In suspensions, the LVR limit for isolates increased from 22.50% as the oil concentration increased. At lower concentrations, a slightly decreasing limit is visible.

The concentrate suspensions showed other correlations. With regard to SPC, there was only a slight influence of oil, whereas there was a clear positive effect with up to 15% oil and then a decline with regard to PPC.

Heating changes the influence of oil on the LVR. The isolates show curves with a maximum at 7.50% or 15% oil, with the exception of PPI at 65 °C. Here, the course of the curve is similar to that observed without heating, as seen with SPC at 65 °C. In contrast, a positive linear relationship is recognizable in SPC at 95 °C. With regard to PPC at 65 °C and 95 °C, the linear correlation is negative.

### 3.2. Networking Factor

**Figure 3:**
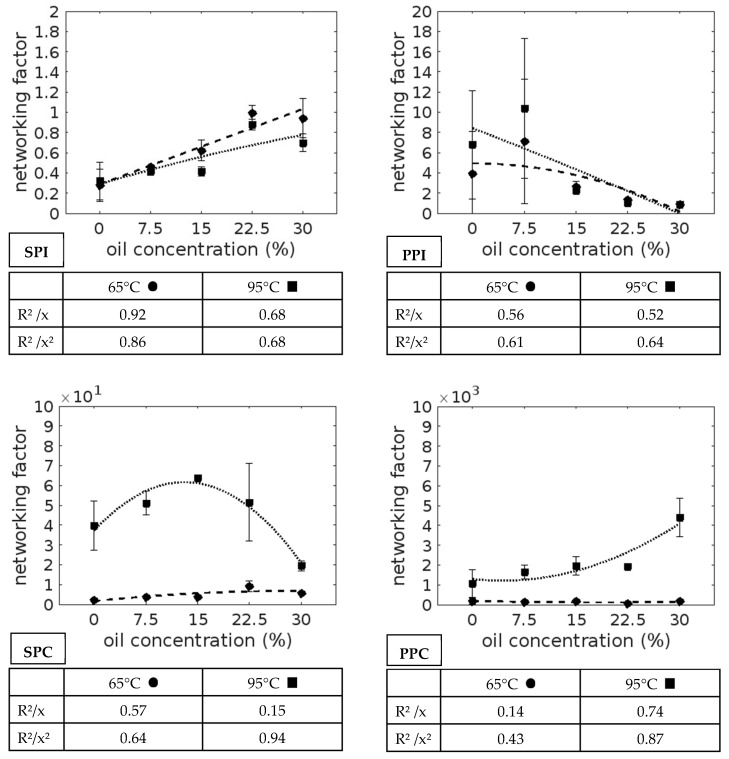
Networking factor. SPI—soy protein isolate, PPI—pea protein isolate, SPC—soy protein concentrate, PPC—pea protein concentrate, 65 °C—after 65 °C heating cycle, 95 °C—after 95 °C heating cycle, R^2^—measure of certainty, x—linear function, x^2^—second-degree polynomial function, line of dashes and dots—trend line without heating cycle, dashed line—trend line after 65 °C heating cycle, dotted line—trend line after 95 °C heating cycle.

In order to better visualize the effect of different heating temperatures, the gel strengths, G′, before and after heating were compared to indicate the degree of cross-linking (Figure 3).

For the concentrates, all values are >1. This indicates that heating consistently results in an increase in the maximum storage modulus, in contrast to the isolates. Particularly for SPI, heating exhibits no discernible effect (all networking factors < 1). Regarding PPI, the sample with 30.00% oil shows a lower storage modulus after heating.

Furthermore, concentrates show higher networking factors compared to isolates, thus indicating why the y-axes differ by a factor of 10 (SPC) and even by a factor of 1000 (PPC). In particular, PPC shows very high networking factors (1061.26 to 4402.20). In addition, for the concentrates, the higher temperature of 95 °C results in higher values than after heating at 65 °C. Thus, a higher temperature results in firmer gels and, likely, increased cross-linking of the proteins.

The influence of the oil concentration on the networking factor varies according to the different samples. With regard to SPI at 65 °C, there is a clear positive linear relationship (R^2^ = 0.92). This means that a higher oil concentration results in a higher networking factor. For PPC at 95 °C, the correlation is almost linear (R^2^ = 0.74), with only a slight increase between 15.00% and 22.50% oil. Therefore, the correlation can also be described somewhat better by a second-degree polynomial trend line (R^2^ = 0.87).

Heating to 95 °C changes the relationship between the oil and the networking factor in SPI. Here, the oil concentration causes the networking factor to increase, up to 22.50% oil, and then decrease. It is likely that the networking factors would continue to decrease as oil concentrations increase. This correlation can also be seen in SPC at 65 °C. However, in SPC at 95 °C, this maximum is already exceeded at 15% oil, and the relationship could be accurately described by a second-degree polynomial function (R^2^ = 0.94). With regard to PPI at 65 °C and 95 °C, it can also be seen that the networking factor increases to a peak at 7.50% oil concentration and then decreases. Oil had little effect on the networking factor in PPC at 65 °C. The values tended to fluctuate around an average value due to the addition of oil.

### 3.3. Analysis of the Microstructe by Scanning Electron Microscopy (SEM) 

**Figure 4:**
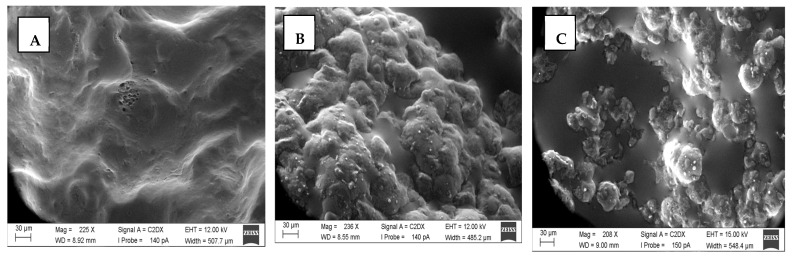
Microstructure of pea protein isolate (95 °C, 60 min). (**A**)—0% oil, (**B**)—15% oil, (**C**)—30% oil.

In addition to the rheological properties of gels, the microstructure provides further insight into gel properties and the role played by proteins and carbohydrates. The following figures (Figure 3 and Figure 4) show the microscopic nature of the PPI and SPI gels. Additional microscopic images can be found in the Supplementary Material.

**Figure 5:**
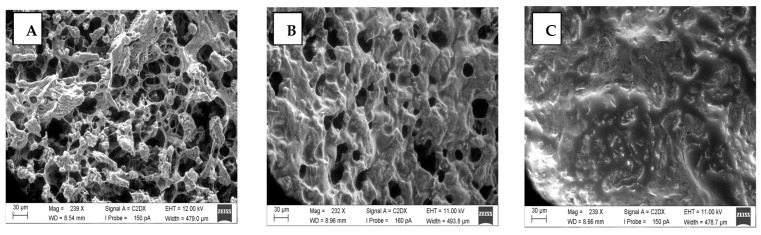
Microstructure of soy protein concentrate (95 °C, 60 min). (**A**)—0% oil, (**B**)—15% oil, (**C**)—30% oil.

Due to its viscous texture, SPI at 0% was not measurable under these conditions (see Supplementary Material for all SEM images). The SEM images show that the proteins formed networks. However, differences between isolates (Figure 4) and concentrates (Figure 5) are recognizable.

Isolates show low cross-linking with apparently larger aggregates, and concentrates show a tighter cross-linking of proteins and carbohydrates. Another striking feature of the isolates is that they appear to form a skin-like structure when measured at the triple point. The images, therefore, likely show the microstructure above the skin-like structure. This is particularly evident for PPI with 0% oil. The formation of the skin-like structure is probably reduced as the oil concentration increases. In general, it appears that the isolates have a more oil-saturated network than the concentrates.

In the concentrates, especially in SPC, the network structures of the proteins and starch become visible. Particularly in the case of concentrates, it can be seen that, as the oil concentration increases, the cavities within the network structures are filled with oil (Figure 5A–C). This is particularly evident in gels with 30% oil content (Figure 5A). At this concentration, it appears as if some oil droplets coalesce.

## 4. Discussion

### 4.1. Viscoelasticity

As the name suggests, a viscoelastic material exhibits mixed behaviors—partly viscous and partly elastic—combining the properties of liquids and solids. The loss factor can be used to determine whether the material is a viscoelastic liquid or gel, as described earlier.

The oil used in this work is a liquid. This means that the firmness can only come from the gelled biopolymers [15]. Therefore, the required protein powder concentration was adjusted to the least gelling concentration (LGC). The LGC is the minimum protein concentration at which a self-supporting gel is formed (see Table A1). All samples show a higher storage module than the loss module or a loss factor below 1 after heating to 65 °C or 95 °C. This means that the least gelling concentration causes viscoelastic gels. This is also the case when oil is added.

Even without heating, the suspensions and emulsions show a viscoelastic gel behavior. The exception is PPC at 22.5% and 30% oil concentrations, with loss factors greater than 1. Yu et al. (2020) also found that a soy protein isolate suspension exhibited viscoelastic behavior, even at 25 °C [16]. The swelling properties of proteins are likely to be sufficient to produce a rheological viscoelastic gel. These swelling characteristics of PPC are probably exhausted via increased oil concentration. Furthermore, these properties depend on various factors, including the macronutrient composition, proportion of hydrophilic and hydrophobic amino acids, spatial structure, etc. [17].

In general, the rheological properties measured here are influenced by various factors. In the following sections, these factors and their influence on the results obtained are analyzed in more detail.

### 4.2. Influence of Macronutrient Components

The isolates and concentrates used here have a different macronutrient composition (see Table 1). This can influence the rheological parameters determined.

The suspensions and emulsions of PPC generally have higher loss factors compared to all other samples. Furthermore, SPI has the highest LVR limits, which could be due to its protein content, as PPC has the lowest protein content and SPI the highest. However, even though SPC contains less protein than PPI, SPC shows lower loss factors than PPI. Thus, there are likely other factors involved. On the one hand, the ability of proteins to swell, bind water/oil, and form/stabilize emulsions is likely to play a role in terms of rheological properties [18]. On the other hand, in addition to proteins, carbohydrates may also exhibit water-binding or swelling properties due to their hydrophilic nature and, therefore, have a positive influence on the gel character [19].

Similar to proteins, carbohydrates can also form gels. The combination of the two biopolymers increases the concentration of gel-forming substances, which can promote gel formation [17,20]. This could explain the higher cross-linking factors of the concentrates compared to the isolates, which is why the y-axes differ by a factor of 10 (SPC) and even by a factor of 1000 (PPC). In particular, PPC has very high cross-linking factors. The network structures in the concentrates are also clearly visible in the SEM images when compared to the isolates. In the case of SPC at 0% oil (Figure 5A), a starch network is also likely visible. However, PPC shows higher networking factors, although it has a lower concentration of biopolymers (protein–carbohydrates) than SPC.

It is probable that the carbohydrates in PPC have superior gelling properties and can interact better with the proteins. The gelation of protein–polysaccharide complexes is influenced by the nature and structure of the polysaccharides, the protein–polysaccharide ratio, and the flexibility, size, and shape of the protein molecule [17,21,22]. Intermolecular and physical interactions, such as electrostatic, hydrogen, or hydrophobic bonds, are responsible for the gelation and interaction of polysaccharides and proteins [23]. For example, polyanion–polycation systems are formed via proteins below their isoelectric point and anionic polysaccharides [24]. Furthermore, the hydrophilic nature of polysaccharides binds a large amount of free water, which may favor disulfide bridge bonds and lead to a more compact gel [19]. However, polysaccharides can also impair gelation. Legume proteins, for example, cannot interact with neutral polysaccharides [25]. Moreover, polysaccharides can also block hydrophobic binding sites [26]. Comparing PPC and SPC reveals that the positive influences in PPC likely outweigh the negative ones, resulting in higher networking factors.

Moreover, legume starches, such as pea starch, exhibit a low gelation temperature. According to Leite et al. (2017), pea starch begins gelling at 58.79 °C [27]. This could explain the difference in cross-linking factors between the isolate and concentrate even at 65 °C. Kornet et al. (2021) also showed that pea protein fractions with higher carbohydrate and lower protein contents could have a higher storage modulus and elasticity than fractions after isoelectric precipitation of the proteins [28].

### 4.3. Influence of Protein Chemistry

In addition to the macronutrient content, protein chemistry also plays an important role with regard to gel properties.

In comparison to the other gels, SPC has lower loss factors after the 95 °C heating cycle. In terms of amino acid composition, SPC contains a higher proportion of sulfur amino acids than the other proteins (see Table 2). This promotes the formation of disulfide bonds and can decrease the loss factor [28]. It is also worth noting that the LVR limits for SPC are lower in comparison. Dreher et al. (2020) found a decreasing LVR limit with increasing protein cross-linking [29]. Likely due to the higher proportion of sulfide-containing amino acids, protein cross-linking through disulfide bridges is more pronounced in SPC, resulting in lower LVR limits.

Apart from this, it is noticeable that the amino acid composition of the different protein powders does not significantly differ from each other. Minor differences, such as a higher glutamic acid content in soy, are relativized when the proportion of all hydrophilic amino acids is taken into account. Furthermore, the techno-functional properties and gel formation are determined not only by the primary structure but also by the quaternary structure and position of the functional groups within the spatial structure [30].

Moreover, the quaternary structure is influenced by the method used to purify the product. According to Boye et al. (2010) and Batista et al. (2005), the purification method of the protein may have a greater influence than the protein concentration [31,32]. It is likely that pre-treating the isolates, particularly SPI, resulted in an unfavorable quaternary structure, resulting in poorer gel formation.

Furthermore, the ability to denature depends not only on the temperature but also on the method used to pretreat or purify the protein powder. For instance, in a study by Li et al. (2007), soy protein concentrate did not show an endothermic peak upon heating from 25 to 150 °C, which was caused by denaturation during production [33]. According to the manufacturer, only PPC is produced in a solely physical way. In the case of the other proteins, the manufacturing processes could have already led to partial denaturation of the proteins and affected the gelling capacity. This may also explain the very high networking factors in PPC upon heating, as the proteins are still present in the native state.

### 4.4. Influence of Heating

In this study, the gels were heat-induced using two different temperatures: 65 °C and 95 °C. Heating changes the rheological properties. Prior to heating, the swelling and emulsifying properties primarily influence the rheological behavior. After heating, emulsion gels are formed, and the rheological parameters are mainly determined via the network created and the way in which the oil phase is incorporated into the network. The microscopic images also show that networks formed.

Heating, especially at 95 °C, led to a decrease in the loss factor, an increase in the LVR limit, and a networking factor > 1. This was only partially the case at 65 °C. According to Chen et al. (2016), increasing temperature increases the aggregation rate of soy globulins. At 85 °C, just a few minutes were enough to form aggregates [34]. Furthermore, the denaturation temperatures of the main protein fractions are 80–88 °C for glycinin and 63–68 °C for beta-conglycinin in soy and 76.6 °C for legumin and 68.5 °C for vicilin in pea [35]. At 65 °C with a 10 min holding time, the proteins likely start to denature, whereas all proteins should be denatured and able to form a better network at 95 °C with a 10 min holding time.

This is not the case with every isolate gel. In general, during the preparation of the suspensions and emulsions, it was observed that isolates had a thicker consistency than concentrates. The soy protein isolate, in particular, formed a highly viscous suspension. This probably affected gel formation, leading to an increase in the loss factor and networking factors < 1. Regarding PPI, the sample with 30.00% oil exhibited a lower storage modulus after heating. The 30% oil content likely exceeded the concentration of oil that could be actively incorporated into the gel network.

### 4.5. Influence of Oil Content

In addition to the two different heating levels, the oil content also varied. Increasing the oil content resulted in different effects, which are discussed below.

As mentioned above, the viscoelastic properties (loss factor and LVR limit) of suspensions and emulsions likely depend on the cold swelling properties (water and oil holding capacity). These are influenced by various factors, such as the macronutrient composition, proportion of hydrophilic and hydrophobic amino acids, spatial structure, etc. [30].

Swelling and viscoelastic properties are affected differently by increasing oil concentration, depending on the raw material used. Positive but small effects can be seen in the case of isolates and soy protein raw materials. The swelling properties are likely not yet fully exploited or are barely affected by increasing oil concentration.

PPC emulsions exhibit different viscoelastic properties, with low to positive effects measured up to oil contents of 7.50% and 15.00%, which then become negative. The negative influence could be explained by the viscous character of sunflower oil, which dominates the rheological properties. This is reinforced by the lack of cold swelling properties and a lower water holding capacity [30,36,37].

Heating partially alters oil’s effect on viscoelastic properties through gel network formation. The effect of oil on the viscoelastic properties of the gels is highly dependent on the used raw material [9,36,38].

In addition, individual trends can be seen in the respective rheological properties (e.g., loss factor, LVR limit, and networking factor) of the same raw material. For example, increasing the oil concentration in SPI at 65 °C leads to better loss and networking factors, but the linear viscoelastic range is only positively influenced up to a specific oil concentration.

This shows that the various measurement parameters should be considered in a differentiated way. The loss factor gives an indication of the ratio of unbound molecules, which consume deformation energy through friction with their environment, to bound molecules, where the deformation energy is stored in the network. The LVR indicates the range in which deformation is irreversible or where the sample structure remains unchanged despite the mechanical energy applied [39]. In this study, the calculated networking factor shows the ratio of the gel’s strength or the intermolecular cohesion of different oil levels to the respective hydrogel [39]. Gel strength is determined by the extent of interactions (hydrogen bonds, hydrophobic interactions, electrostatic interactions, and covalent bonds) between different structural elements (biopolymer matrix–biopolymer matrix, and biopolymer matrix–oil droplet and oil droplet–oil droplet emulsion) [37,40].

With regard to the positive effects of oil, it acts as an active filler matrix and reinforces the gel network. Protein-stabilized or coated oil droplets act as active fillers when incorporated into a protein gel matrix. They are mechanically bound to the gel matrix and support the gel’s strength [9,41]. Reinforcement is a result of strong interactions, a low loss factor due to the good integration of the molecules, and a high LVR due to shear stability [9,39]. If heating has a positive effect on the viscoelastic properties, it is likely that partial denaturation has exposed hydrophobic sites that can better interact with the oil [42,43,44].

According to other authors, positive correlations were also found with regard to increasing the oil concentration and gel strength. In a study by Gu et al. (2009), the strength of heat-induced soya protein emulsion gels increased as oil concentrations increased from 5 to 20% [13]. Moreover, a positive effect of oil (0–15%) on gel properties was also measured by Silva et al. (2019), who explored gels made from soy protein isolate, pea protein isolate, and mixtures of these with micellar casein after a heating and holding temperature of 90 °C [45].

In addition to positive effects, some gels exhibit only a slight influence of oil on viscoelastic properties and network factors. Similarly, in a study by Kornet et al. (2022), it was found that increasing the oil content of pea emulsion gels at a neutral pH exhibited little effect on gel strength [14]. The oil droplet likely interacts only slightly with the protein matrix, as the hydrophobic regions of the proteins may be directed toward the oil phase and, therefore, may not be available for gel network formation [41,46,47].

Increasing oil concentrations can also exert negative effects on rheological properties. Here, the oil acts as an inactive filler and weakens the network [9]. The inactive fillers have a low chemical affinity for the gel matrix. Therefore, they act as small holes in the network and weaken the gel [9,41]. In addition, at specific oil concentrations, a crowding effect of filler particles can occur among high volume fractions and particle packing limits [48]. This can also be seen in the microscopic images (Figure 5C), as 30% coalescence is visible.

The oil concentration at which this phenomenon can occur depends on the properties of the raw material. This also explains the polynomial curves, in which the oil has an initial positive effect on the viscoelastic properties and then a negative effect.

There are also correlations that suggest that a negative to low impact is initially perceived and then becomes positive. A specific concentration must be reached before the oil can positively affect the viscoelastic properties and act as an active filler. Prior to this concentration, the oil acts as an inactive filler, creating holes in the matrix, and the elasticity of the gel decreases as the number of holes in the matrix increases [47,49].

## 5. Conclusions

The gelling properties of different soy and pea protein powders were determined in this study by measuring and comparing the rheological parameters and the microstructure of the gels. The influence of raw material (macronutrient content and amino acid composition), heating temperature, and oil concentration was also analyzed.

According to the results, the rheological and microstructural properties of emulsion gels are influenced by a number of factors. The texturizing potential for emulsion gels of legume protein powders is, therefore, individual for each commercially available product. However, based on these results, the carbohydrate content and the purification method of the legumes used as a texturing agent seem to play an important role. The level of the storage modulus characterizes the strength. The results show that the carbohydrate content contributes significantly to increasing the storage modulus and the strength. This is illustrated by the differences between the isolates and concentrates tested, with the concentrates showing more stable and tighter cross-linked gels. The influence of a higher heating temperature on the rheological properties was also more pronounced in the concentrates, probably due to the higher carbohydrate content. This indicates that proteins from more sustainable sources may also be suitable for use in emulsion gel production

Furthermore, the effect of oil on gel properties is mainly dependent on the properties of the protein powder used. In addition to concentration, oil properties also play an important role, which could be further analyzed in future studies.

Animal products, such as cooked sausages or cheese, are structurally emulsion gels. This structure is formed via animal proteins. Emulsion gels are, therefore, a suitable form to mimic such products. Further studies that specifically address the product context are needed to explore the potential of legume proteins in relation to alternative products.

## Figures and Tables

**Table 1:** Macronutrient amount of the protein powders in g/100 g of product, according to the manufacturer.

Nutrients	SPI ^1^	PPI ^2^	SPC ^3^	PPC ^4^
Carbohydrate	2.30	0.80	3.26	18.50
Fiber	<1.00	2.40	2.17	5.78
Fat	0.50	4.00	0.54	6.94
Protein	92.50	81.70	73.91	47.58
Salt	1.24	3.70	1.09	0.01

SPI—soy protein isolate, PPI—pea protein isolate, SPC—soy protein concentrate, PPC—pea protein concentrate, ^1^—EUROSOY, ^2^—Cosucra, ^3^—EUROSOY, ^4^—Herba ingredients.

**Table 2:** Amino acid composition of the protein powders in g/100 g of protein, according to the manufacturer.

Nutrients	SPI ^1^	PPI ^2^	SPC ^3^	PPC ^4^
Alanin	4.27	4.30	4.49	4.42
Arginin	7.74	8.70	7.80	8.60
Aspartic acid	11.67	11.50	11.52	12.18
Cysteine	1.14	1.00	2.72	1.36
Glutamic acid	20.79	16.80	19.79	17.04
Glycine	4.50	4.10	4.19	4.36
Histidine	3.23	2.50	2.64	2.52
Isoleucine	5.31	4.50	4.96	4.40
Leucine	8.09	8.40	8.25	7.69
Lysine	7.05	7.20	6.63	8.05
Methionine	1.29	1.10	1.01	1.01
Phenylalanine	5.54	5.50	5.47	5.41
Proline	4.85	4.50	5.53	4.42
Serine	4.16	5.30	4.94	5.12
Threonine	3.47	3.90	3.92	3.94
Tryptophan	1.85	1.00	0.80	0.94
Tyrosine	2.66	3.80	3.29	3.77
Valine	5.54	5.00	5.18	4.76

SPI—soy protein isolate, PPI—pea protein isolate, SPC—soy protein concentrate, PPC—pea protein concentrate, ^1^—EUROSOY, ^2^—Cosucra, ^3^—EUROSOY, ^4^—Herba ingredients.

**Table 3:** Microscope settings of different gels.

	0% Oil Concentration					15% Oil Concentration					30% Oil Concentration				
	M (X)	EHT (kV)	WD (X)	I Probe (pA)	Width (μm)	M (X)	EHT (kV)	WD (X)	I Probe (pA)	Width (μm)	M (X)	EHT (kV)	WD (X)	I Probe (pA)	Width (μm)
PPI	225	12.00	8.96	140	507.70	236	12.00	8.55	140	485.20	206	15.00	9.00	150	548.40
SPC	239	12.00	8.54	150	479	232	11.00	8.96	160	493.80	239	11.00	8,66	150	478.7

PPI—pea protein isolate, SPC—soy protein concentrate, M = magnification, EHT = extra high tension, WD = working distance, I Probe = electron beam width, width = width of the measured sample section, (X) = no dimension.

## Data Availability

The original contributions presented in the study are included in the article/Supplementary Material, further inquiries can be directed to the corresponding author.

## Supplementary Materials

The following supporting information can be downloaded at https://www.mdpi.com/article/10.3390/foods13233875/s1: Figure S1: Amplitude sweeps of soy protein isolate; Figure S2: Amplitude sweeps of pea protein isolate; Figure S3: Amplitude sweeps of soy protein concentrate; Figure S4: Amplitude sweeps of pea protein concentrate; Figure S5: Microstructure of soy protein isolate (95 °C, 60 min); Figure S6: Microstructure of pea protein concentrate (95 °C, 60 min); Table S1: Microscope settings of different gels.

## Appendix A

### Least Gelling Concentration (LGC)

The LGC method of Sathe and Salunkhe (1981) was used in this work in a slightly different way [11]. Protein suspensions containing 2%, 4%, 5%, 10%, and 20% (*w*/*w*) of the protein powder were prepared. This allowed a rough classification of the gelation point. In order to be able to determine the LGC more precisely, smaller increments in percentage were chosen (1% (*w*/*w*) steps). The 1% steps were again subdivided into 0.25%, 0.50%, and 0.75% (*w*/*w*) steps. A 25 mL protein suspension was prepared in a 50 mL sealable tube using an Ultra Turrax IKA T18 basic (Staufen, Germany) at 7000 rpm for one minute. The tubes were then placed in a water bath at 95 °C for 60 min. Immediately after heating, the tubes were cooled at 5–7 °C overnight in a cold storage room. The LGC is the minimum protein concentration at which a self-supporting gel is formed. This is assessed by inverting the tube, and if the gel holds for three minutes, it is said to be a self-supporting gel. Table A1 shows the least gelling concentrations of the legume proteins.

**Table A1:** Least gelling concentration of legume protein powders.

	SPI	PPI	SPC	PPC
LGC (%)	9.50	12.00	11.50	10.75

SPI—soy protein isolate, PPI—pea protein isolate, SPC—soy protein concentrate, PPC—pea protein concentrate.
