# Supplementary material for: The Viscoelastic Behavior of Legume Protein Emulsion Gels—The Effect of Heating Temperature and Oil Content on Viscoelasticity, the Degree of Networking, and the Microstructure
Source: Foods. 2024 Nov 29;13(23):3875. doi: 10.3390/foods13233875 (PMC11640714; doi:10.3390/foods13233875)
Supplement: Supplementary file 1 [file foods-13-03875-s001.zip › foods-3297957-supplementary.pdf]

Supplementary Material

Rheological properties – Oscillation curve of G' and G''

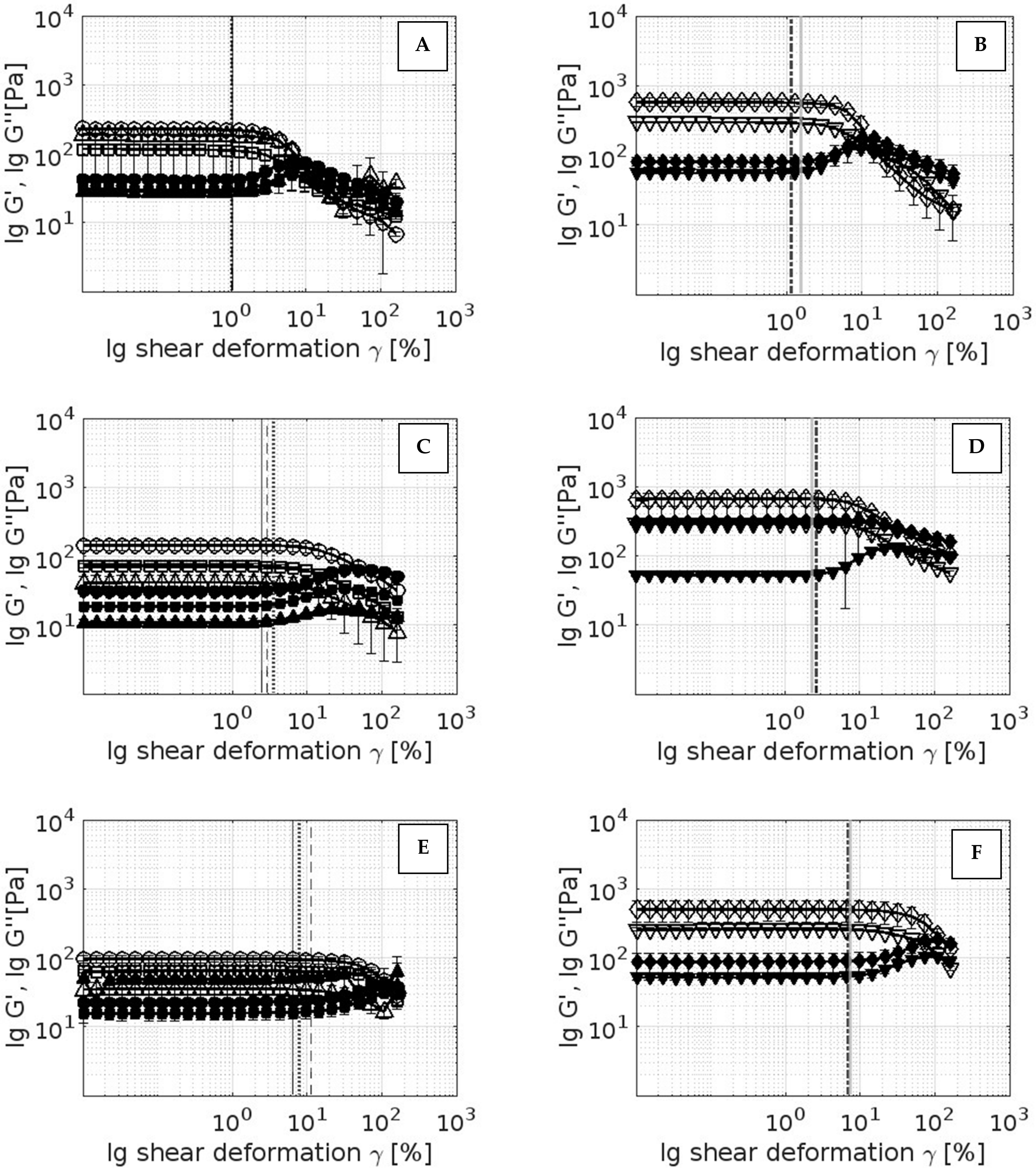

Figure S1. Amplitude sweeps of soy protein isolate. A= before heating, B = before heating, C= after heating at 65°C, D= after heating at 65°C, E= after heating at 95°C, F= after heating at 95 °C. Empty symbols = storage module, filled symbols = loss module. triangle = 0% oil, square= 7,5% oil, circle = 15% oil, inverted triangle = 22,5% oil diamond = 30% oil. Error bars = standard deviation. Constant line = LVR-Limit of 0% oil, dashed line = LVR-Limit of 7.5% oil, dotted line = LVR-Limit of 15% oil, dashed dotted line = LVR-Limit of 22.5% oil, constant line (grey) = LVR-limit of 30% oil

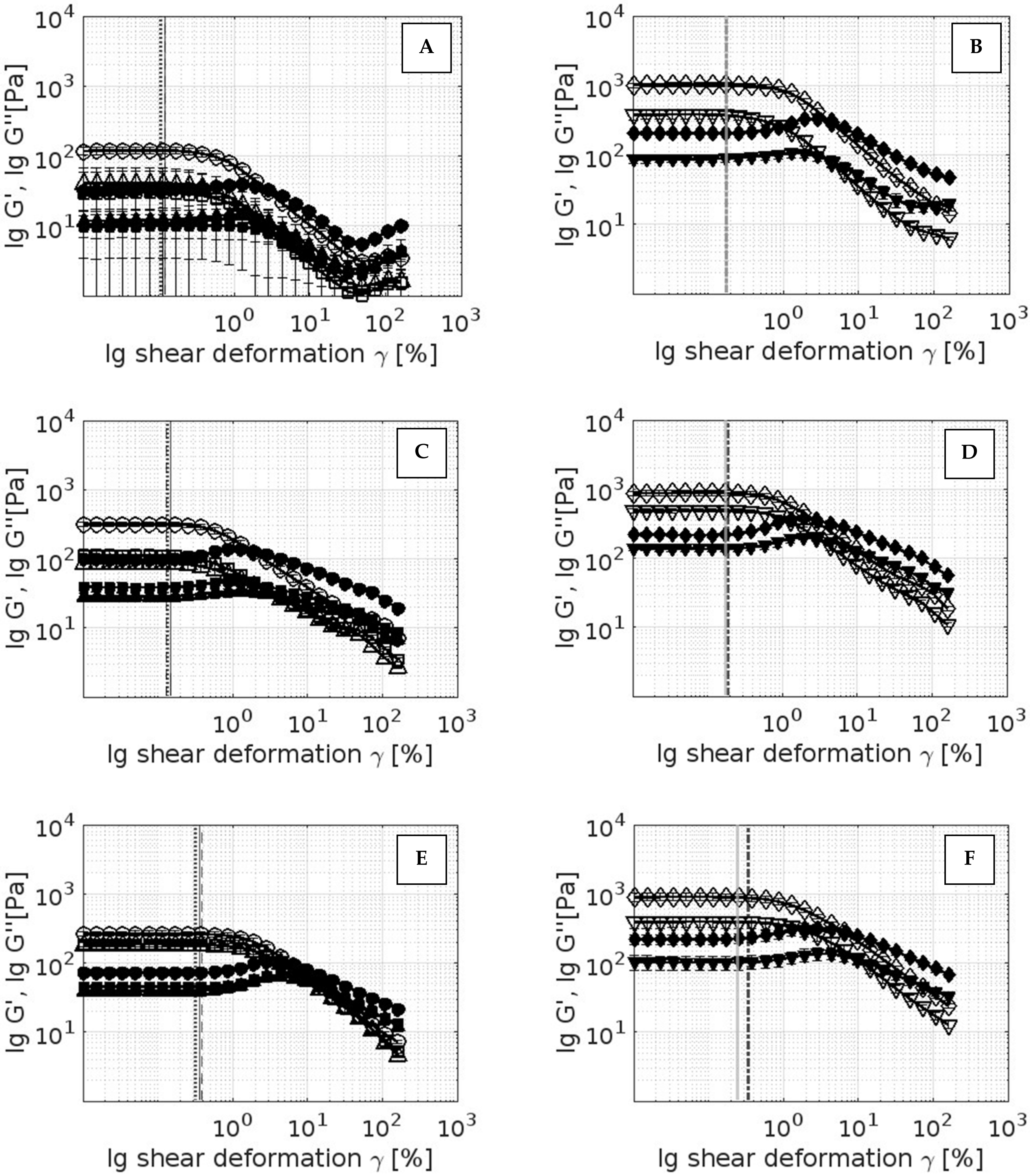

Figure S2. Amplitude sweeps of pea protein isolate. A= before heating, B = before heating, C= after heating at 65°C, D= after heating at 65°C, E= after heating at 95°C, F= after heating at 95 °C. Empty symbols = storage module, filled symbols = loss module. triangle = 0% oil, square= 7,5% oil, circle = 15% oil, inverted triangle = 22,5% oil diamond = 30% oil. Error bars = standard deviation. Constant line = LVR-Limit of 0% oil, dashed line = LVR-Limit of 7.5% oil, dotted line = LVR-Limit of 15% oil, dashed dotted line = LVR-Limit of 22.5% oil, constant line (grey) = LVR-limit of 30% oil

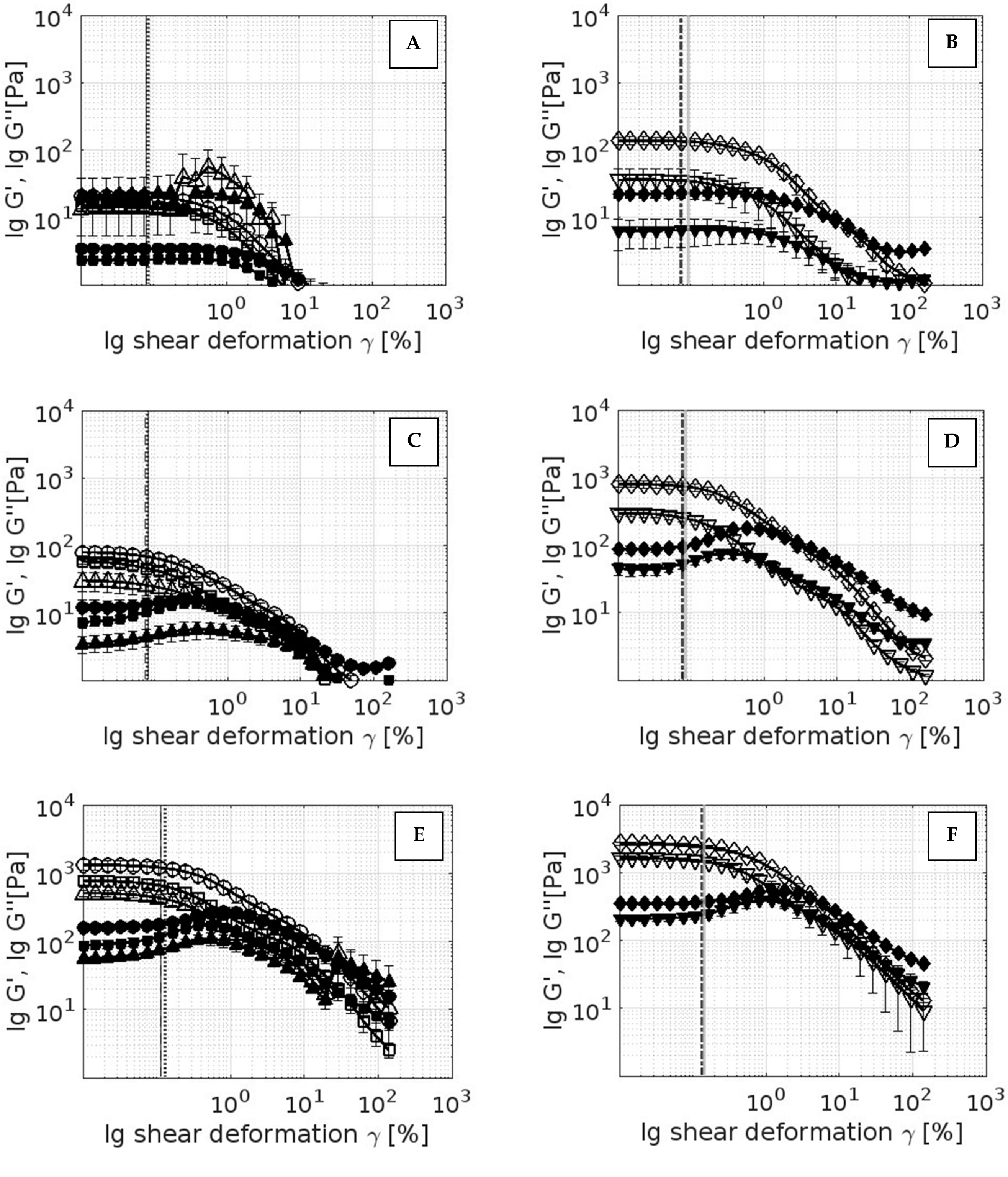

Figure S3. Amplitude sweeps of soy protein concentrate. A= before heating, B = before heating, C= after heating at 65°C, D= after heating at 65°C, E= after heating at 95°C, F= after heating at 95 °C. Empty symbols = storage module, filled symbols = loss module. triangle = 0% oil, square= 7,5% oil, circle = 15% oil, inverted triangle = 22,5% oil diamond = 30% oil. Error bars = standard deviation. Constant line = LVR-Limit of 0% oil, dashed line = LVR-Limit of 7.5% oil, dotted line = LVR- Limit of 15% oil, dashed dotted line = LVR-Limit of 22.5% oil, constant line (grey) = LVR-limit of 30% oil

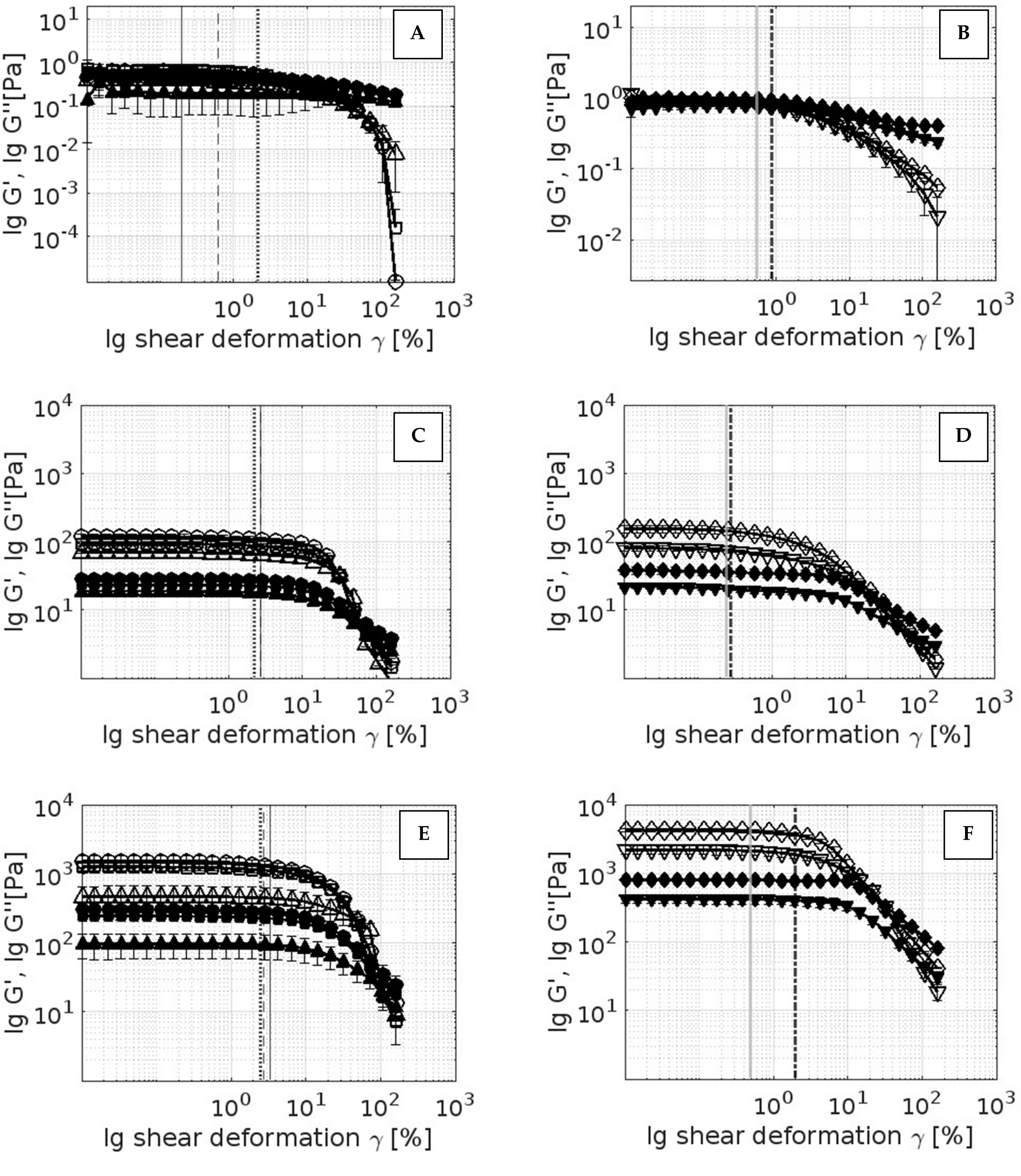

Figure S4. Amplitude sweeps of pea protein concentrate. A= before heating, B = before heating, C= after heating at 65°C, D= after heating at 65°C, E= after heating at 95°C, F= after heating at 95 °C. Empty symbols = storage module, filled symbols = loss module. triangle = 0% oil, square= 7,5% oil, circle = 15% oil, inverted triangle = 22,5% oil diamond = 30% oil. Error bars = standard deviation. Constant line = LVR-Limit of 0% oil, dashed line = LVR-Limit of 7.5% oil, dotted line = LVR-Limit of 15% oil, dashed dotted line = LVR-Limit of 22.5% oil, constant line (grey) = LVR-limit of 30% oil

Microscopy properties

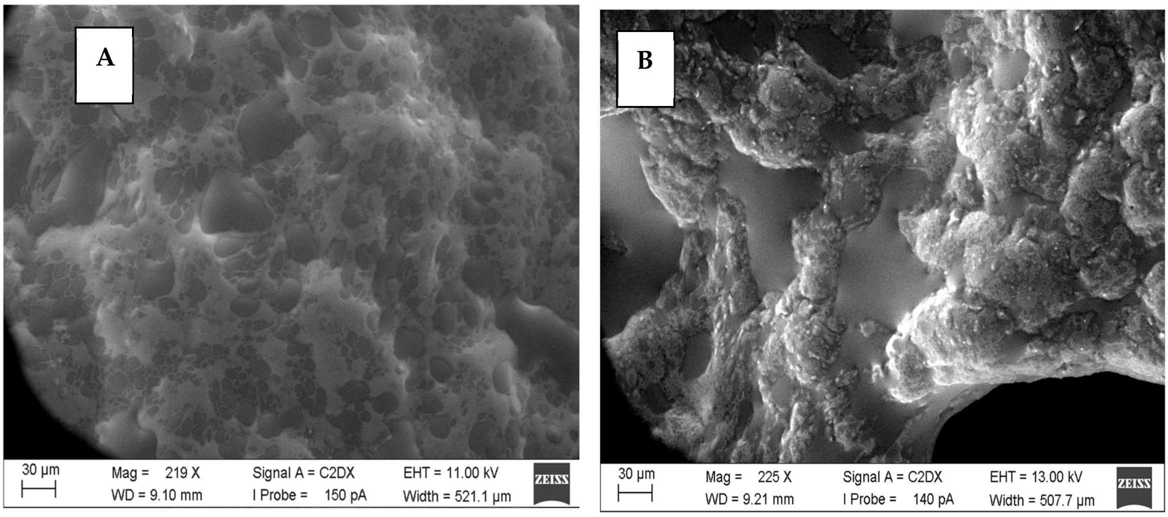

Figure S5. Microstructure of soy protein isolate (95°C, 60min). A = 15% oil, B = 30% oil

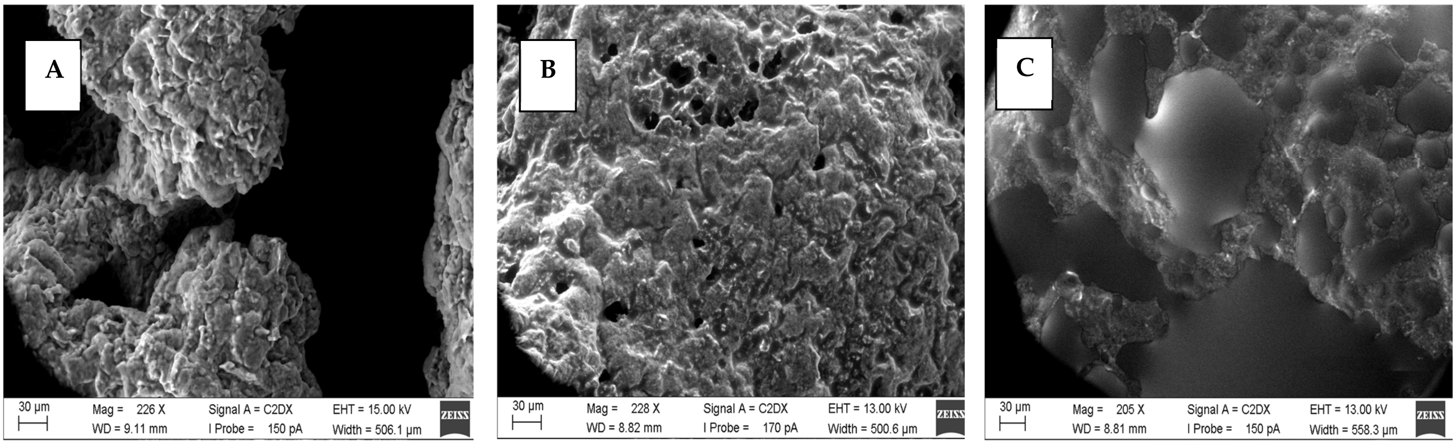

Figure S6. Microstructure of pea protein concentrate (95°C,60min). A = 0% oil, B = 15% oil, C = 30% oil

Table S1. Microscope settings of different gels

	**0% oil concentration**					**15% oil concentration**					**30% oil concentration**				
	M (X)	EHT (kV)	WD (X)	I Probe (pA)	Width (µm)	M (X)	EHT (kV)	WD (X)	I Probe (pA)	Width (µm)	M (X)	EHT (kV)	WD (X)	I Probe (pA)	Width (µm)
**SPI**	x	x	x	x	x	219	11.00	9.10	150	521.10	225	13.00	9.21	140	507.70
**PPC**	226	15.00	9.11	150	506.1	228	13.00	8.82	170	500.60	205	13.00	8.81	150	558.3

SPI—soy protein isolate, PPC—pea protein concentrate, M = Magnification, EHT = Extra High Tension, WD = Working Distance, I Probe = Electron beam width, Width = Width of the measured sample section, (X) = no dimension
